# Giant splenic artery aneurysm in a pregnant patient: a case report and literature review

**DOI:** 10.1002/ccr3.1000

**Published:** 2017-06-01

**Authors:** Elie Creidi, Antoine El Asmar, Rawad Abou Zahr, Ziad El Rassi

**Affiliations:** ^1^ General Surgery ResidentSaint Georges Hospital University Medical CenterBeirutLebanon; ^2^ Faculty of MedicineUniversity of BalamandBeirutLebanon; ^3^ Urology ResidentSaint Georges Hospital University Medical CenterBeirutLebanon; ^4^ Department of Clinical SurgeryGeneral and Digestive Surgery – Oncologic SurgerySaint Georges Hospital University Medical CenterBeirutLebanon

**Keywords:** Aneurysms in pregnant patient, giant splenic artery aneurysm

## Abstract

Pregnancy and giant splenic artery aneurysms should be addressed in a way to achieve optimal results for the mother and the fetus. In our case, the need for immediate intervention, with minimal risk, made open aneurysmectomy and distal splenopancreatectomy, the ideal approach to undertake.

## Introduction

Splenic artery aneurysms [SAA] are an infrequently diagnosed entity. This condition is more common in women 1 and four times more in multiparous women 2 due to the physiologic changes in pregnancy. The hormonal changes seem to affect aneurysmal dilatation 3. Pregnancy appears to carry a higher burden of complications, with rupture and hemorrhage being more frequent and thus a higher rate of maternal and fetal death 4. To note, two‐third of SAA rupture occurs in the third trimester 5. SAA rarely exceeds 3 cm 5 and most studies consider aneurysms ≥5 cm as giant splenic artery aneurysms 6 and those are usually symptomatic with a higher risk of rupture 7.

## Case Report

This is the case of a 29‐year‐old female patient, G2P1001, at 12 weeks + 4 days, referred to us for management of giant splenic artery aneurysm. History goes back to 11 months prior to presentation when the patient had severe left upper quadrant pain during the 8th month of her first pregnancy. Her pain was then attributed to uterine contractions and thus she was delivered by Caesarean section with no further investigations carried out at that time.

**Figure 1:**
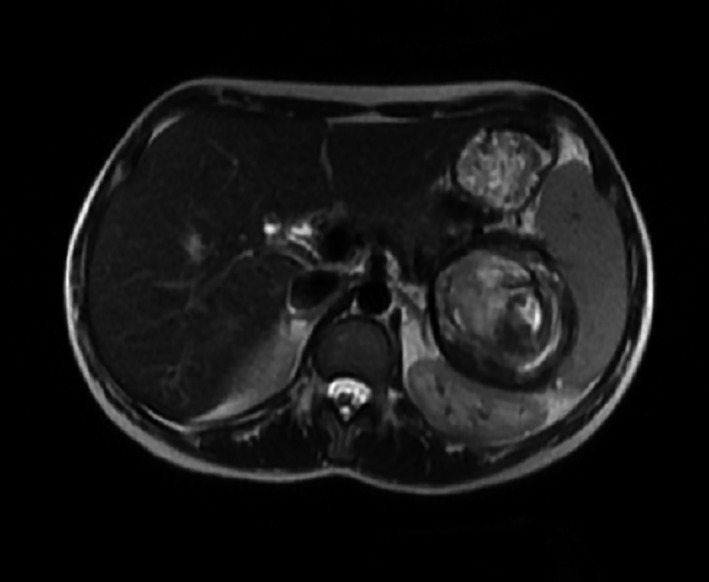
Axial cut on MRI showing a 7.5 × 7 × 6.5 cm aneurysm in the distal portion of the splenic artery with an intramural thrombus, abutting the splenic hilum.

**Figure 2:**
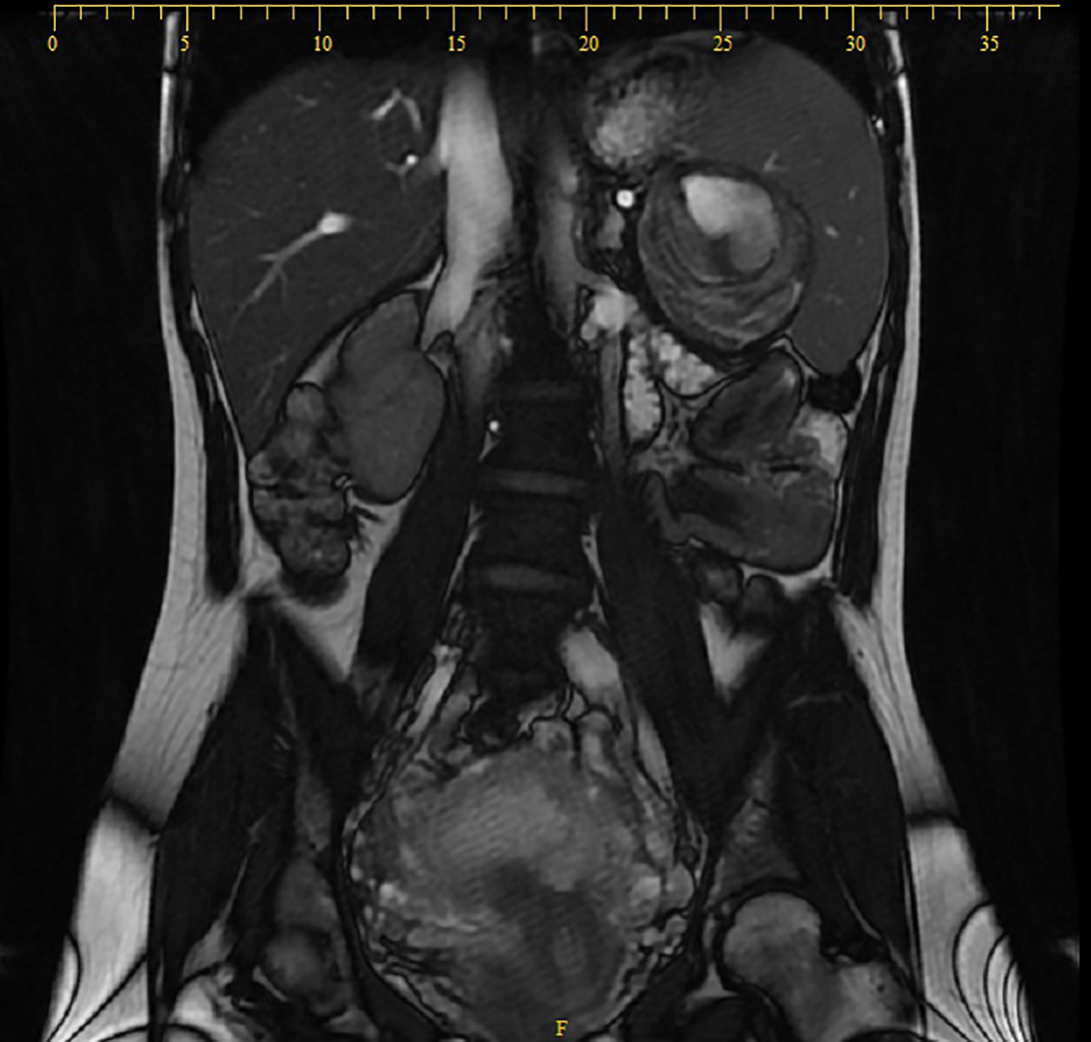
Coronal cut on MRI showing a 7.5 × 7 × 6.5 cm aneurysm in the distal portion of the splenic artery with an intramural thrombus, abutting the splenic hilum.

During her subsequent pregnancy and few days before presentation, she developed the same left upper quadrant pain. Ultrasound was performed showing: enlarged spleen 15.5 cm, well‐defined rounded heterogeneous lesion of 7.6 × 6.8 cm in favor of a splenic artery aneurysm. MRI was performed for better evaluation (Figs 1 and 2), revealing a single aneurysm 7.5 × 7 × 6.5 cm in the distal portion of the splenic artery with an intramural thrombus, abutting the splenic hilum.

Laboratory studies performed were normal.

At this point, open aneurysmectomy was decided for the following reasons: the large size of the aneurysm, its location in the distal third of the splenic artery, the extensive involvement of the splenic hilum, the potential need for a formal splenectomy, the fact that our patient was pregnant, and the potential risk of rupture in endovascular repair.

**Figure 3:**
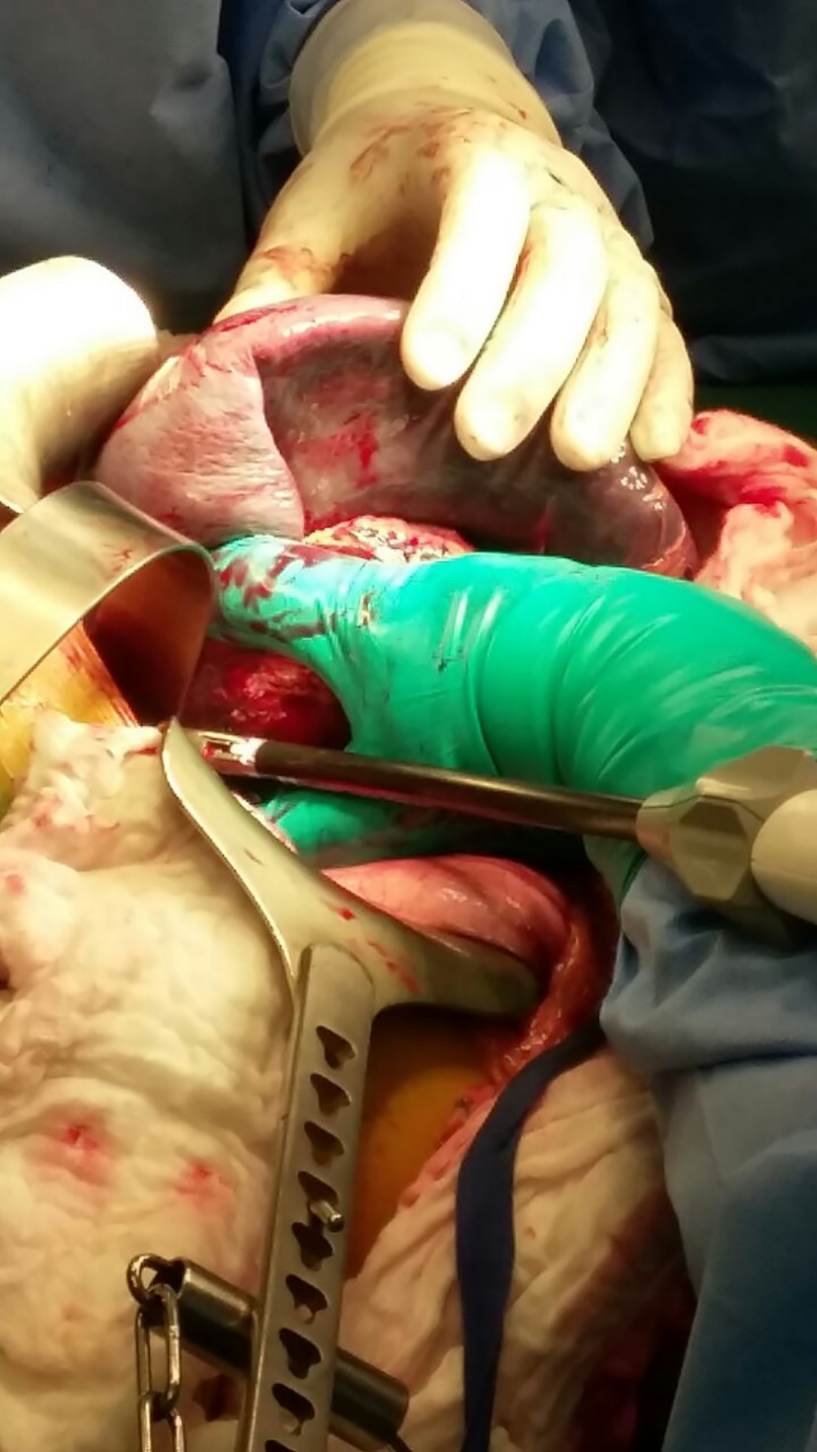
Intraoperative image of giant SAA involving the splenic hilum.

**Figure 4:**
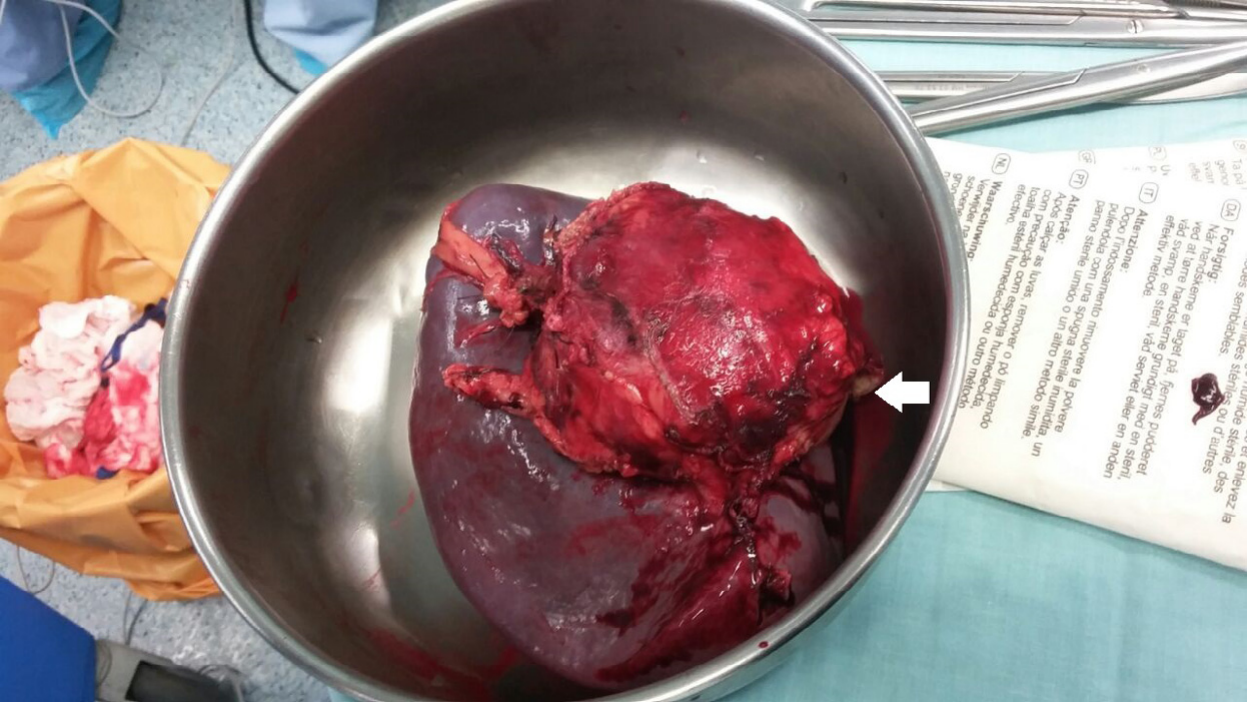
Intraoperative image of the specimen after removal, showing the spleen, the aneurysm, and resected pancreatic tail [white arrow].

**Figure 5:**
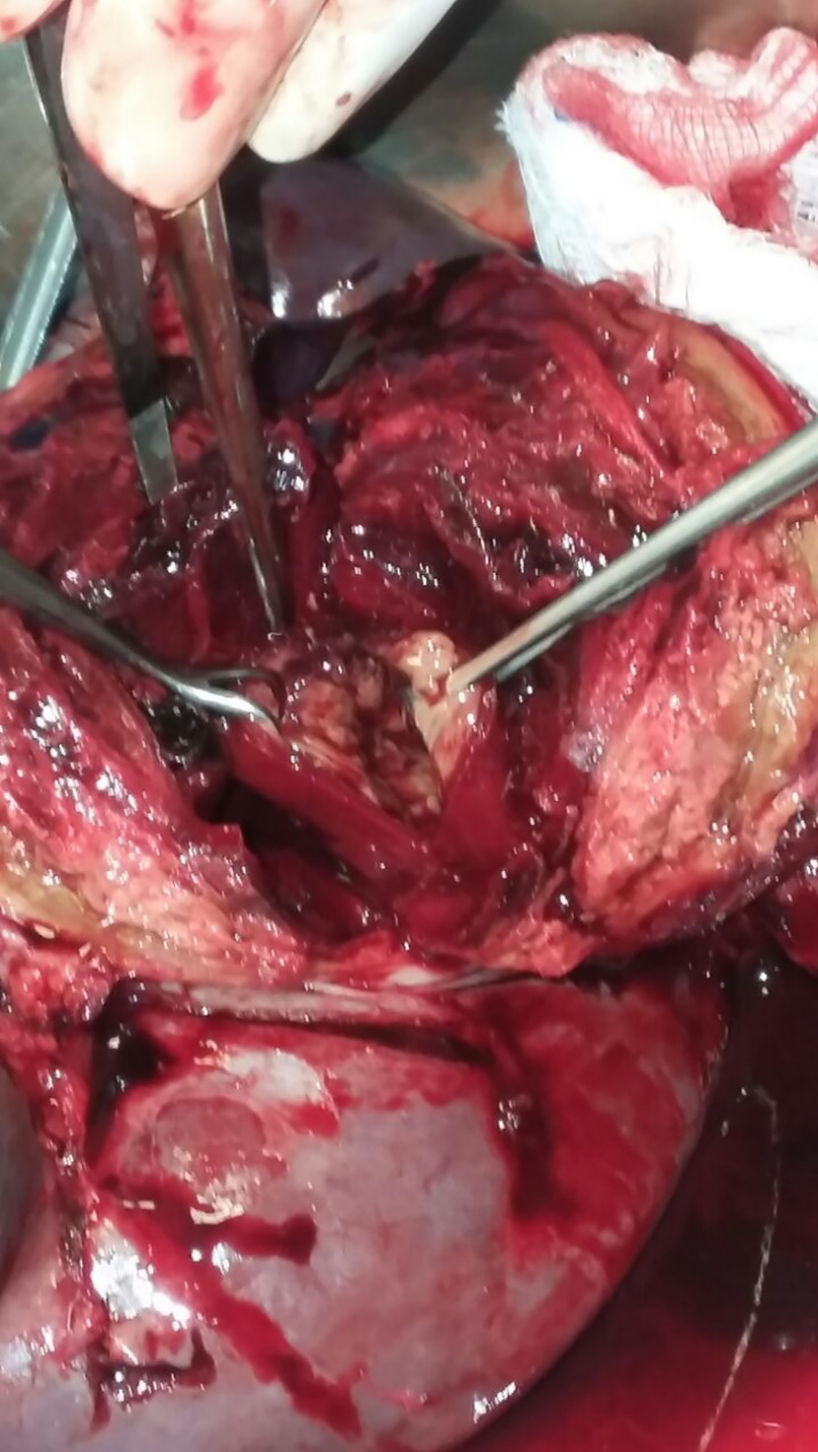
Intraoperative image of the specimen when opened, showing the extensive thrombus within.

A left subcostal incision performed, entering the lesser sac by division of the gastrocolic ligament and liberation of the splenic flexure. The aneurysm could be palpated over the distal pancreas and splenic hilum, dissection and control of the splenic artery proximally. Dissection of the gastrosplenic ligament and ligation of the short gastric vessels. The aneurysm was adherent to the pancreatic tail, and exhibiting a severe desmoplastic reaction. Thus, a distal pancreatectomy was warranted. Ligation and division of the splenic artery proximally and distal pancreatectomy was thus performed. Ligation and division of the splenic vein, liberation of spleen from the surrounding structures (Fig. 3), and removal of the specimen en bloc was carried out (Figs 4 and 5).

No perioperative complications were encountered.

Obstetrical ultrasound performed to assess fetal well‐being on day 1 post‐op and 2 months post‐op were both normal.

Final pathology showed aneurysm of the splenic artery, filled with fresh thrombus, associated with compression and involvement of the pancreatic tissue and secondary focal atrophy.

## Discussion

Splenic artery aneurysms are the 3rd most encountered intraabdominal aneurysms although the true incidence may be underestimated because most cases are asymptomatic 8. It is more common in females with a ratio of 4:1, whereas giant SAAs are 1.78 times more common in male patients 9.

Hypertension and subsequent atherosclerosis, female gender, pregnancy, multiparity, cirrhosis, portal hypertension, and liver transplant are important risk factors for SAA, with pregnancy being the most common cause for rupture 8. The 3rd trimester carries the highest risk 10. It is thought that increased cardiac output, increased splenic artery outflow and hormonal changes accompanied with pregnancy are responsible for the development, progression and rupture of SAA 11.

In pregnancy, and similar to the rest of the population, most SAA are asymptomatic. The increase use of ultrasound in pregnancy contributes to the higher incidence of SAA in that population 12.

In the 20% of patients who are symptomatic, epigastric and left upper quadrant abdominal pain are the most described symptoms 9. During pregnancy, high degree of suspicion is required because SAA can easily be misdiagnosed as uterine rupture or placental abruption 12. Complications of SAA include spontaneous rupture, fistulization into neighboring organs, or duct of Wirsung and arteriovenous fistula between the portal vein and the aneurysm, and rupture being the most fatal one 10. Spontaneous rupture is more frequent in pregnant women and even more whenever a giant SAA is present 9.

In the absence of a clear consensus, treatment is currently performed in patients with symptomatic SAAs or asymptomatic SAAs ≥2 cm, those who have portal hypertension or those who are candidates for liver transplant 9, 10. The use of open, laparoscopic, or endovascular approach is nowadays the mainstay of treatment. Open abdominal surgery consists of aneurysmectomy with or without splenectomy and distal pancreatectomy depending on the location of the aneurysm. Laparoscopic excision is performed for smaller lesions with minimal risk of rupture. Endovascular entails embolization or stent graft in uncomplicated low‐risk cases 8, 9, 10.

Pregnancy is regarded as an absolute indication for proactive management 11, 12 and minimally invasive techniques such as transcatheter embolization, percutaneous angiographic embolization, or laparoscopic ligation are encouraged if the size and location are appropriate 12. In case of rupture, resuscitation and cessation of bleeding are necessary to save the mother and fetus. This is usually carried out by a Caesarian section laparotomy and splenectomy or splenopancreatectomy 12.

The case presented above was complicated in terms of aneurysmal size [giant], location [hilar], and nearby organs involvement [pancreas]. The fact that our patient was also pregnant posed an additional challenge as to the type of management required. The need for immediate intervention, with minimal risk on the mother and fetus, made open aneurysmectomy and distal splenopancreatectomy the ideal approach to undertake.

Splenic artery aneurysm is a rare entity. When diagnosed and treatment is warranted, the ideal method for intervention should be case tailored. Pregnancy and giant aneurysms should be addressed in a more careful method to achieve optimal end results for both the mother and the fetus.

## Authorship

AEA: did the literature review, corrected the article and assisted in the surgical intervention. EC: wrote the article and organized the figures, tables and legends. RAZ: did the data collection and obtained the patient's consent. ZER: performed the operation and reviewed the written article.

## Conflict of Interest

The authors declare no potential conflict of interest.

## References

[1] Arca, M. J. , M. Gagner , B. T. Heniford , T. M. Sullivan , and E. G. Beven . 1999 Splenic artery aneurysms: methods of laparoscopic repair. J. Vasc. Surg. 30:184–188. 10394168 10.1016/s0741-5214(99)70190-4

[2] Dave, S. P. , E. D. Reis , A. Hossain , P. J. Taub , M. D. Kerstein , and L. H. Hollier . 2000 Splenic artery aneurysm in the 1990s. Ann. Vasc. Surg. 14:223–229. 10796953 10.1007/s100169910039

[3] Mattar, S. G. , and A. B. Lumsden . 1995 The management of splenic artery aneurysms: experience with 23 cases. Am. J. Surg. 169:580–584. 7771620 10.1016/s0002-9610(99)80225-6

[4] Lang, W. , D. Strobel , E. Beinder , and M. Raab . 2002 Surgery of a splenic artery aneurysm during pregnancy. Eur. J. Obstet. Gynecol. Reprod. Biol. 102:215–216. 11950496 10.1016/s0301-2115(01)00608-x

[5] Trastek, V. F. , P. C. Pairolero , J. W. Joyce , L. H. Hollier , and P. E. Bernatz . 1982 Splenic artery aneurysms. Surgery 91:694–699. 7079972

[6] Ho, M. F. , Y. C. Chan , and S. W. Cheng . 2013 Successful endovascular management of giant splenic artery aneurysms. Vascular 21:317–322. 23493275 10.1177/1708538113478744

[7] Yadav, S. , P. Sharma , P. K. Singh , S. Punia , P. Desai , A. K. Anjan , et al. 2012 Giant splenic artery aneurysm: a rare but potentially catastrophic surgical challenge. Int. J. Surg. Case Rep. 3:533–536. 22902799 10.1016/j.ijscr.2012.06.010 PMC3437397

[8] Miao, Y. D. , and B. Ye . 2013 Intragastric rupture of splenic artery aneurysms: three case reports and literature review. Pak. J. Med. Sci. 29:656–659. 24353598 PMC3809230

[9] Akbulut, S. , and E. Otan . 2015 Management of giant splenic artery aneurysm: comprehensive literature review. Medicine (Baltimore) 94:e1016. 26166071 10.1097/MD.0000000000001016 PMC4504560

[10] Uyar, ?. S. , F. F. Okur , B. Akp?nar , F. Abac?lar , V. Yurtman , V. ?ahin , et al. 2013 A giant splenic artery aneurysm: a case report. Turkish Journal of Thoracic and Cardiovascular Surgery 21:799–802.

[11] Bakhos, C. T. , B. C. McIntosh , F. A. Nukta , P. N. Fiedler , R. W. Denatale , T. F. Sweeney , et al. 2007 Staged arterial embolization and surgical resection of a giant splenic artery aneurysm. Annals of vascular surgery 21:208‐210. 17349364 10.1016/j.avsg.2007.01.005

[12] Sadat, U. , O. Dar , S. Walsh , and K. Varty . 2008 Splenic artery aneurism in pregnancy – a systematic review. Int. J. Surg. 6:261–265. 17869597 10.1016/j.ijsu.2007.08.002

